# Neurovascular hand symptoms in relation to cold exposure in northern Sweden: a population-based study

**DOI:** 10.1007/s00420-017-1221-3

**Published:** 2017-04-11

**Authors:** Albin Stjernbrandt, Bodil Björ, Martin Andersson, Lage Burström, Ingrid Liljelind, Tohr Nilsson, Ronnie Lundström, Jens Wahlström

**Affiliations:** 1 Department of Public Health and Clinical Medicine, Umeå University, 901 87 Umeå, Sweden; 2 Department of Radiation Sciences, Umeå University, 901 87 Umeå, Sweden; 3 Occupational and Environmental Medicine, University Hospital of Umeå, 901 85 Umeå, Sweden

**Keywords:** Cold exposure, Sweden, Hand, Frostbite, Cold sensitivity, Raynaud’s phenomenon

## Abstract

**Purpose:**

To describe the self-reported ambient cold exposure in northern Sweden and to relate the level of cumulative cold exposure to the occurrence of sensory and vascular hand symptoms. We hypothesize that cold exposure is positively related to reporting such symptoms.

**Methods:**

A questionnaire about cold exposure and related symptoms was sent out to 35,144 subjects aged 18–70 years and living in northern Sweden.

**Results:**

A total of 12,627 out of 35,144 subjects returned the questionnaire (response rate 35.9%). Subjects living in the rural alpine areas reported more extensive cold exposure both during work and leisure time compared to the urbanized coastal regions. Frostbite in the hands was present in 11.4% of men and 7.1% of women, cold sensitivity was present in 9.7 and 14.4%, and Raynaud’s phenomenon was present in 11.0% of men and 14.0% of women. There was a positive association between cumulative cold exposure and neurovascular hand symptoms.

**Conclusion:**

The present study demonstrates that the cold environment in northern Sweden might be an underestimated health risk. Our hypothesis that cold exposure is positively related to reporting of neurovascular hand symptoms was supported by our findings. In addition, such symptoms were common not only in conjunction with an overt cold injury. Our results warrant further study on pathophysiological mechanisms and suggest the need for confirmatory prevalence studies to support national public health planning.

**Electronic supplementary material:**

The online version of this article (doi:10.1007/s00420-017-1221-3) contains supplementary material, which is available to authorized users.

## Introduction

Living in a cold climate is associated with numerous adverse health effects (Hassi et al. 2005; Makinen and Hassi 2009). A number of studies have documented increased wintertime morbidity and mortality, especially in the respiratory and cardiovascular systems (Gasparrini et al. 2015; Raatikka et al. 2007; The Eurowinter Group 1997). Cold exposure can also cause specific injuries to the extremities, such as frostbite and chilblains, and is associated with long-term neurological and vascular dysfunction (Carlsson et al. 2016; Imray and Castellani 2012). Different pathophysiological mechanisms of such cold injuries have been studied and it has been shown that reduced tissue temperature due to ambient cold exposure can lead to several detrimental vascular effects, such as vasoconstriction, ischemia-induced endothelial damage, hemoconcentration, and the creation of free radicals (Thomas and Oakley 2002). Some of these effects are believed to contribute to vascular hand symptoms, such as Raynaud’s phenomenon (Garner et al. 2015). When it comes to the peripheral nerves, apart from direct damage from cooling, the ischemic vascular effects mentioned above can also cause nerve dysfunction through effects on the vasa nervorum, the small arteries that supply blood flow to the peripheral nerves (Thomas and Oakley 2002).

Cold sensitivity has previously been defined as an exaggerated or abnormal reaction to cold exposure causing discomfort or the avoidance of cold (Kay 1985), and it has often been studied in conjunction with hand injuries, such as digital amputations or hand-arm vibration injury (Carlsson et al. 2010; Lithell et al. 1997). The pathophysiological mechanisms in cold sensitivity might involve both neurological and vascular aspects (Hope et al. 2014; Thomas and Oakley 2002). To the authors’ knowledge, cold sensitivity has not previously been investigated in large-scale population-based studies. However, one Swedish study included a small sample of subjects from the general population (*N* = 94), (Carlsson et al. 2010).

Regarding exposure to cold, temperatures at or below 10 °C are defined as ambient cold exposure in occupational health standards (International Organization for Standardization 2008). The experience of being cold can also be defined from a subjective standpoint regardless of the ambient temperature (Makinen and Hassi 2002). Cold exposure can occur during both work and leisure time and is often associated with aggravating environmental conditions, such as wind, rain, or snow (Keim et al. 2002). Indoor work with cold storage, contact with cold objects, and immersion in cold water can also contribute (Baldus et al. 2012). In addition, the effects of ambient cold exposure are modified by individual factors, such as sex, age, nutritional status, pre-existing diseases, medication, thermal insulation of clothing, and activity level (Raatikka et al. 2007). Swedish national statistics report that 22% of working men and 11% of working women in Sweden are occupationally exposed to cold for more than one quarter of their working hours (Swedish Work Environment Authority 2014). Living in the northern parts of Sweden can also result in significant leisure-time cold exposure, but national statistics for such exposure are not available. Large-scale, questionnaire-based studies of cold-related health effects have been undertaken in Finland (Makinen et al. 2009; Nayha et al. 2011; Raatikka et al. 2007), but such studies are lacking in Sweden.

The aim of the present study was to describe the self-reported ambient cold exposure in northern Sweden and to relate the level of cumulative cold exposure to the reported occurrence of sensory and vascular hand symptoms. We hypothesize that cold exposure is positively related to reporting such symptoms.

## Methods

### Participants and data collection

The Cold and Health in Northern Sweden (CHINS) studies were launched in early 2015 and consist of several questionnaire-based surveys, of which this paper represents the first publication. The present study had a descriptive cross-sectional design and focused on the characteristics of the baseline cohort and the occurrence of hand symptoms. It was conducted in the four northernmost counties in Sweden: Norrbotten, Västerbotten, Västernorrland, and Jämtland. The study region holds a population of approximately 880,000 people and is located between 62°N and 69°N latitude with a mixed subarctic and temperate climate. The data collection was initiated on the fifth of February 2015 and was ended on the fifth of May 2015. The mean monthly temperature during the study period ranged from −9.4 to 4.9 °C, with the lowest temperatures recorded in the alpine regions and the highest in the coastal regions (Swedish Metrological and Hydrological Institute 2015).

**Fig. 1:**
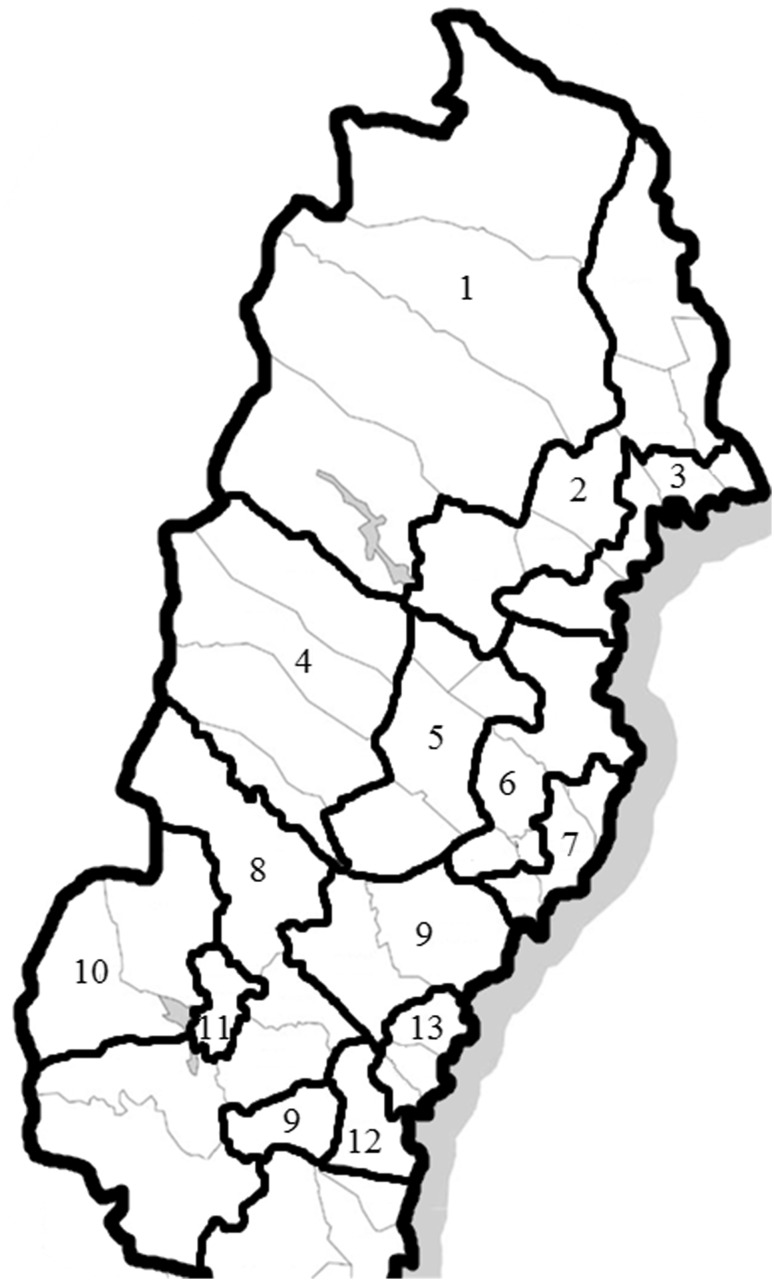
Climate zones of northern Sweden. The data collection was stratified according to the 13 climate zones that are shown with numbers in the figure. The ninth climate zone is made up of two different areas. *Thick black lines* show the border of each climate zone, and *thin grey lines* depict the different counties

The data collection was performed by means of a mail-in survey on a sample of men and women between 18 and 70 years of age living in the study area who were drawn from the national Swedish population register. The study region was subdivided into 13 climate zones (Fig. 1) depending on the mean annual temperature (The Swedish Horticultural Society 2015), and a fixed number of questionnaires was allocated to each of these climate zones (*N* = 1,350 per zone, *N* = 17,550 in total), after which the remaining number (*N* = 17,594) was proportionally distributed according to the population size in each climate zone. This semi-randomized approach was chosen to ensure a sufficient sample from sparsely populated areas, where cold exposure can be profound. One questionnaire per individual was sent by mail, without subsequent reminders.

### Study design

The study questionnaire included 38 items and was designed by a ten-person team consisting of three occupational and environmental physicians, two ergonomists, two occupational and environmental hygienists and three engineers. The questionnaire collected baseline data on demographic and anthropometric variables in the form of place of residence, sex, age, height, and weight. The daily use of tobacco, either cigarettes or snuff, was asked about. Other questions asked if the study participants had been diagnosed by a physician for any of the following: hypertension, angina pectoris, myocardial infarction, stroke, asthma, chronic obstructive pulmonary disease, diabetes mellitus, joint disorders, or migraine. The occupation of the study participants was collected in free text and then coded in accordance with the International Standard Classification of Occupations (International Labour Organization 2012). Geographical location was determined by postal code and stratified into 44 municipalities that were then grouped together to form three broad categories—coastal, inland, and alpine.

We defined cold exposure as being outside during the winter time study period or in cold environments indoors. The participants were asked to specify their exposure during work and leisure time by grading their exposure on two separate cold exposure variables:

1. “During work I am exposed to outdoor or cold environments.”
2. “During leisure time I am exposed to outdoor or cold environments.”

The answers were graded on a numerical scale in whole numbers ranging from 1 (“do not agree”) to 10 (“fully agree”). In the analyses, data were categorized into a four categories, where 1 was categorized as “none”, 2–4 as “low”, 5–7 as “medium”, and 8–10 as “high” exposure. For logistic regression analyses, the occupational and leisure-time exposure variables were summed to form a cumulative exposure variable ranging from 2 (“do not agree”) to 20 (“fully agree”). This variable was then divided into quartiles. Through this approach, the contribution of cold exposure was assumed to be of similar importance regardless of whether it occurred during work or leisure time.

Frostbite was categorized according to location (hands, feet, or face) and as first degree (white spots), second degree (blisters), or third degree (blood-filled blisters), inspired by a previously described classification system (Imray et al. 2009). For questions regarding the sensory and vascular function of the hands, a previously tested descriptive scale was used (Hagberg et al. 2008). Questions were posed, such as: “Have you experienced decreased touch perception in your hand?” The answers were given on a four-grade scale as “none”, “insignificant”, “somewhat”, or “a lot”. Answering “somewhat” or “a lot” was considered a positive response. Cold sensitivity was determined with the statement, “I am over-sensitive to cold”, to which the study participant could answer on a fixed numerical scale ranging from 1 (“do not agree”) to 10 (“agree completely”), where an answer of 4 or more was considered a positive response. The occurrence of Raynaud’s phenomenon was investigated through a single item question, “Does one or more of your fingers turn white (as shown on picture) when exposed to moisture or cold?”, and this was supported by a standardized color chart that has previously been shown to increase the diagnostic specificity (Negro et al. 2008).

### Statistical analysis

The statistical dependence of the two cold exposure variables was measured using Spearman’s rank correlation coefficient. For the relationship between the cumulative exposure variable (leisure time and occupational cold exposure) and different symptoms, odds ratios with 95% confidence intervals were calculated using logistic regression. *P* values ≤ 0.05 were considered statistically significant. All statistical analyses were performed with IBM SPSS Statistics for Windows (version 23.0, IBM Corporation, Armonk, NY, USA).

## Results

### Participants

A total of 35,144 questionnaires were sent out, of which 34,822 reached their intended addressees and 12,627 were filled in and returned for a final response rate of 35.9%. An analysis of non-responders revealed minor demographical differences in terms of sex, age, and home district as compared to the responders (Online Resource 1). The response rate increased with age and was slightly higher among women.

The final study population consisted of 45.5% men and 54.5% women, and the mean age was 52 and 50 years, respectively. Men had a higher body mass index than women [26.7 (standard deviation 4.0) kg/m^2^ versus 25.6 (standard deviation 4.9) kg/m^2^]. Daily smoking was reported by 7.2% of men and 9.8% of women, and daily use of snuff was reported by 23.2 and 8.5%, respectively. The anthropometric data and occurrence of diseases are presented in detail in Table 1.

**Table 1:** Study group characteristics

	Men			Women		
	Mean	SD	Range	Mean	SD	Range
Age (years)	52	14.6	18–70	50	14.6	18–70
Height (cm)	179	6.8	145–206	166	6.2	116–194
Weight (kg)	86	14.2	45–191	70	13.9	32–177
BMI (kg/m^2^)	26.7	4.0	13.9–62.6^a^	25.6	4.9	13.1–73.7^a^

	*N*	%	*N*	%
Daily smoking	407	7.2	663	9.8
Daily use of snuff	1315	23.2	576	8.5
Hypertension	1515	27.2	1454	21.6
Angina pectoris	180	3.2	117	1.7
Myocardial infarction	207	3.7	81	1.2
Stroke	129	2.2	79	1.2
Asthma	591	10.5	907	13.5
Chronic obstructive pulmonary disease	75	1.3	76	1.1
Diabetes mellitus	374	6.6	256	3.8
Joint disease	387	6.9	806	12.0
Migraine	288	5.1	794	11.8
Frostbite in the hands	644	11.4	486	7.1
Frostbite in the feet	604	10.7	664	9.8
Frostbite in the face	1665	29.6	1415	20.8
Reduced sensitivity to touch	449	7.9	482	7.1
Reduced sensitivity to warmth	338	6.0	328	4.8
Reduced sensitivity to cold	318	5.6	254	3.8
Swollen fingers when cold	518	9.2	1050	15.5
Cold sensitivity	544	9.7	973	14.4
Pain or discomfort in hands when cold	1145	20.4	1784	26.5
Raynaud’s phenomenon	620	11.0	949	14.0

Anthropometric data, tobacco habits, diseases, and symptoms in the study group

*SD* standard deviation, *BMI* body mass index

^a^The range is affected by outliers. When these were excluded (BMI <15 or >40, men *N* = 53; women *N* = 89), the mean BMI was 26.6 for men and 25.4 for women

Both occupational and leisure-time cold exposures were higher among men than women, and high levels of cold exposure were inversely proportional to age (Table 2). Study participants living in alpine areas more frequently reported high cold exposure during work (17.3%) and leisure time (37.0%) compared to those living in the coastal regions (10.7 and 22.9%, respectively). The highest occupational cold exposure was reported among those working in the armed forces, where 75.8% reported a high cold exposure during work. Other highly cold-exposed occupational groups were skilled agricultural, forestry, and fishery workers (67.0%); crafts and related trades workers (e.g., construction workers) (29.7%); plant and machine operators and assemblers (26.1%); and elementary occupations (e.g., miners and refuse workers) (22.5%). These five occupational groups were all most common in alpine areas and less common in coastal areas (data not shown). The correlation between occupational and leisure-time cold exposure variables was rather low, but statistically significant (*r*_s_ = 0.27, *p* value < 0.01). Detailed exposure data can be found in Table 2.

**Table 2:** Occupational and leisure-time cold exposure

	Occupational cold exposure				Leisure-time cold exposure			
	None (NRS 1)	Low (NRS 2–4)	Medium (NRS 5–7)	High (NRS 8–10)	None (NRS 1)	Low (NRS 2–4)	Medium (NRS 5–7)	High (NRS 8–10)
	*N* (%)	*N* (%)	*N* (%)	*N* (%)	*N* (%)	*N* (%)	*N* (%)	*N* (%)
Sex								
Male	2488 (45.7)	981 (18.0)	989 (18.2)	988 (18.1)	480 (8.6)	1208 (21.5)	2256 (40.2)	1663 (29.7)
Female	4525 (69.2)	853 (13.0)	597 (9.1)	563 (8.6)	986 (14.7)	1598 (23.9)	2448 (36.5)	1666 (24.9)
Age (years)								
18–31	908 (51.3)	312 (17.6)	279 (15.8)	270 (15.3)	181 (10.2)	428 (24.1)	688 (38.7)	481 (27.1)
32–44	1181 (55.9)	346 (16.4)	292 (13.8)	293 (13.9)	157 (7.4)	459 (21.6)	829 (39.1)	677 (31.9)
45–57	1875 (54.9)	595 (17.4)	473 (13.9)	472 (13.8)	326 (9.5)	735 (21.5)	1361 (39.8)	996 (29.1)
58–70	3049 (65.0)	581 (12.4)	542 (11.6)	516 (11.0)	802 (16.1)	1184 (23.7)	1826 (36.6)	1175 (23.6)
Region								
Alpine	1289 (49.8)	434 (16.8)	416 (16.1)	449 (17.3)	234 (8.8)	445 (16.8)	995 (37.5)	982 (37.0)
Inland	1787 (57.4)	463 (14.9)	434 (13.9)	428 (13.8)	371 (11.6)	683 (21.3)	1282 (39.9)	876 (27.3)
Coastal	3937 (62.7)	937 (14.9)	736 (11.7)	674 (10.7)	861 (13.4)	1678 (26.1)	2427 (37.7)	1471 (22.9)
Occupation								
Armed forces occupations	1 (3.1)	2 (6.3)	5 (15.6)	24 (75.8)	0 (0)	6 (19.4)	9 (29.0)	16 (51.6)
Managers	305 (68.7)	83 (18.7)	38 (8.6)	18 (4.1)	32 (7.2)	83 (18.6)	186 (41.6)	146 (32.7)
Professionals	1242 (61.8)	335 (16.7)	203 (10.1)	230 (11.4)	157 (7.8)	483 (24.0)	774 (38.5)	596 (29.7)
Technicians and associate professionals	687 (58.9)	220 (18.9)	147 (12.6)	113 (9.7)	79 (6.8)	259 (22.3)	491 (42.2)	334 (28.7)
Clerical support workers	765 (75.5)	112 (11.1)	67 (6.6)	69 (6.8)	111 (10.9)	299 (29.5)	379 (37.3)	226 (22.3)
Service and sales workers	974 (54.1)	305 (16.9)	275 (15.3)	248 (13.8)	214 (11.9)	399 (22.2)	698 (38.8)	490 (27.2)
Skilled agricultural, forestry, and fishery workers	18 (8.5)	15 (7.1)	37 (17.5)	142 (67.0)	16 (7.5)	20 (9.3)	62 (29.0)	116 (54.2)
Crafts and related trades workers	151 (23.3)	120 (18.5)	184 (28.4)	192 (29.7)	27 (4.2)	125 (19.3)	300 (46.4)	194 (30.0)
Plant and machine operators and assemblers	180 (24.3)	163 (22.0)	204 (27.6)	193 (26.1)	51 (6.9)	130 (17.6)	309 (41.8)	250 (33.8)
Elementary occupations	125 (43.3)	56 (19.4)	43 (14.9)	65 (22.5)	43 (14.9)	87 (30.1)	90 (31.1)	69 (23.9)
Self-employed	86 (37.6)	42 (18.3)	52 (22.7)	49 (21.4)	15 (6.6)	45 (19.7)	94 (41.0)	75 (32.8)
Students	433 (65.3)	123 (18.6)	69 (10.4)	38 (5.7)	102 (15.2)	184 (27.5)	247 (36.9)	136 (20.3)
Unemployed	–	–	–	–	47 (19.8)	62 (26.2)	69 (29.1)	59 (24.9)
Parental leave	–	–	–	–	2 (3.1)	11 (16.9)	29 (44.6)	23 (35.4)
Sick leave	–	–	–	–	35 (20.8)	37 (22.0)	58 (34.5)	38 (22.6)
Retired	–	–	–	–	437 (19.8)	493 (22.4)	782 (35.5)	490 (22.3)

Self-estimated occupational and leisure-time cold exposure was reported on a ten-point numerical rating scale (NRS) and then separately categorized into four groups ranging from low to high exposures. The exposure was then stratified according to sex, age, region, and occupation. For study subjects that were unemployed, retired, or on parental or sick leave, occupational cold exposure data have been omitted. Data are presented as numbers with percentage within brackets

*NRS* numerical rating scale

Ever-occurrence of frostbite in the hands was reported by 11.4% of men and 7.1% of women, while frostbite in the feet was reported by 10.7% of men and 9.8% of women. Facial frostbite was more common at 29.6% in men and 20.8% in women. Second- and third-degree frostbite was present in 0.7–2.1% of men and 0.1–1.0% in women depending on whether it had occurred in the hands, feet, or face. Sensory hand symptoms in the form of decreased perception of touch, warmth, or cold were reported by 5.6–7.9% of men and 3.8–7.1% of women depending on the modality of perception. Certain symptoms occurred specifically in conjunction with cold exposure, such as swollen fingers (9.2% of men and 15.5% of women) and pain or discomfort in the hands (20.4% of men and 26.5% of women). Being cold sensitive was reported by 9.7% of men and 14.4% of women, and Raynaud’s phenomenon was reported by 11.0% of men and 14.0% of women. Logistic regression revealed an association between frostbite in the hands and being cold sensitive (men OR 5.02, 95% CI 3.65–6.90; women OR 5.63, 95% CI 4.31–7.37) as well as reporting Raynaud’s phenomenon (men OR 5.99, 95% CI 4.94–7.27; women OR 6.59, 95% CI 5.42–8.01). High cumulative cold exposure was also positively related to the reporting of cold sensitivity (men OR 1.41, 95% CI 1.08–1.85; women 1.77, 95% CI 1.42–2.20) and Raynaud’s phenomenon (men OR 1.37, 95% CI 1.07–1.76; women OR 1.90, 95% CI 1.51–2.39) (Table 3).

**Table 3:** Association between cumulative cold exposure and symptoms

		Cumulative cold exposure							
		First exposure quartile (2–5)		Second exposure quartile (6–8)		Third exposure quartile (9–11)		Forth exposure quartile (12–20)	
		*N*	OR (95% CI)	*N*	OR (95% CI)	*N*	OR (95% CI)	*N*	OR (95% CI)
Frostbite in hands	♂	81	1 (–)	123	1.18 (0.88–1.58)	157	**1.73 (1.31–2.30)** ^a^	246	**2.29 (1.76–2.99)** ^a^
	♀	85	1 (–)	143	**1.78 (1.35–2.34)** ^a^	152	**2.61 (1.98–3.43)** ^a^	82	**2.40 (1.75–3.28)** ^a^
Frostbite in feet	♂	71	1 (–)	129	**1.43 (1.06–1.94)** ^a^	163	**2.10 (1.57–2.81)** ^a^	213	**2.23 (1.69–2.96)** ^a^
	♀	113	1 (–)	211	**2.02 (1.59–2.56)** ^a^	195	**2.56 (2.01–3.26)** ^a^	119	**2.71 (2.07–3.56)** ^a^
Frostbite in face	♂	185	1 (–)	361	**1.67 (1.37–2.04)** ^a^	430	**2.47 (2.03–3.01)** ^a^	606	**3.02 (2.50–3.65)** ^a^
	♀	284	1 (–)	462	**1.85 (1.57–2.18)** ^a^	394	**2.20 (1.86–2.61)** ^a^	211	**1.99 (1.63–2.42)** ^a^
Reduced sensitivity to touch	♂	64	1 (–)	85	1.02 (0.73–1.43)	91	1.23 (0.88–1.71)	179	**2.05 (1.52–2.75)** ^a^
	♀	118	1 (–)	126	1.09 (0.84–1.42)	108	1.26 (0.96–1.65)	92	**1.93 (1.45–2.57)** ^a^
Reduced sensitivity to warmth	♂	57	1 (–)	69	0.93 (0.65–1.33)	60	0.89 (0.62–1.29)	131	**1.64 (1.19–2.26)** ^a^
	♀	67	1 (–)	79	1.21 (0.87–1.69)	88	**1.83 (1.32–2.54)** ^a^	68	**2.49 (1.76–3.52)** ^a^
Reduced sensitivity to cold	♂	49	1 (–)	58	0.90 (0.61–1.33)	67	1.17 (0.81–1.71)	116	**1.68 (1.19–2.37)** ^a^
	♀	61	1 (–)	66	1.10 (0.77–1.57)	54	1.20 (0.83–1.75)	43	**1.68 (1.13–2.51)** ^a^
Swollen fingers when cold	♂	68	1 (–)	108	1.24 (0.90–1.69)	129	**1.69 (1.24–2.29)** ^a^	187	**2.02 (1.51–2.69)** ^a^
	♀	236	1 (–)	306	**1.38 (1.15–1.66)** ^a^	290	**1.83 (1.52–2.20)** ^a^	172	**1.88 (1.52–2.33)** ^a^
Cold sensitivity	♂	86	1 (–)	126	1.14 (0.85–1.51)	131	**1.33 (1.00–1.77)** ^a^	170	**1.41 (1.08–1.85)** ^a^
	♀	239	1 (–)	263	1.15 (0.95–1.39)	251	**1.52 (1.26–1.84)** ^a^	164	**1.77 (1.42–2.20)** ^a^
Pain or discomfort in hands when cold	♂	168	1 (–)	251	1.18 (0.95–1.46)	270	**1.46 (1.18–1.80)** ^a^	389	**1.77 (1.45–2.17)** ^a^
	♀	410	1 (–)	528	**1.42 (1.23–1.65)** ^a^	462	**1.74 (1.50–2.04)** ^a^	289	**1.97 (1.65–2.35)** ^a^
Raynaud’s phenomenon	♂	103	1 (–)	135	1.01 (0.77–1.32)	138	1.16 (0.88–1.51)	198	**1.37 (1.07–1.76)** ^a^
	♀	198	1 (–)	303	**1.66 (1.37–2.00)** ^a^	250	**1.85 (1.52–2.26)** ^a^	147	**1.90 (1.51–2.39)** ^a^

Self-estimated occupational and leisure-time cold exposure, reported on two separate ten-point numerical rating scales (NRS), has been added together to form a cumulative measurement of cold exposure ranging from 2 to 20. The data were subsequently divided into quartiles, and the first quartile is set as the reference. Associations are presented as odds ratios (OR) with 95% confidence intervals (95% CI)

*NRS* numerical rating scale, *OR* odds ratio, *CI* confidence interval

^a^Bold values indicate odds ratios with significant 95% confidence intervals

## Discussion

The present study shows that cold exposure is common in northern Sweden, both during work and leisure time. However, the correlation between leisure time and occupational exposure is rather low (*r*_s_ = 0.27, *p* value < 0.01), indicating that the study subjects could be highly exposed in one situation but not in the other. In addition, the exposure to cold is generally greater among men than women and is more pronounced in the rural alpine regions as compared to the urbanized coastal regions, which can be explained by certain outdoor occupations, such as forestry and farming being more common, but also due to outdoor leisure-time activities being more popular in these regions. In addition, the lowest mean temperatures in the study area were found in the alpine regions. Taken together, both behavioral and climatic factors contribute to intensive cold exposure in these areas.

Neurovascular hand symptoms, such as reduced sensory perception, increased cold sensitivity, and Raynaud’s phenomenon, were frequently reported and were generally associated with a higher cumulative cold exposure, on a population level. These findings suggest that cold exposure, even in the absence of overt cold injury, such as frostbite or chill blains, might affect the sensory and vascular function of the hands. In addition, previous frostbite to the hands was strongly associated with both cold sensitivity and Raynaud’s phenomenon, which might indicate a common pathophysiological mechanism for these conditions. We hypothesize that cold exposure might cause neurovascular dysfunction in exposed body parts, such as the hands, and give rise to the aforementioned symptoms. There is some, although limited, laboratory (Hope et al. 2014) and epidemiological (Block and Sequeira 2001; Mackiewicz and Piskorz 1977) data to support this concept. Even though cold exposure was generally more pronounced among men, cold-related symptoms were more frequently reported by women. This might be interpreted as a particular susceptibility among women, and this warrants further research.

Previous studies have reported that men are generally more exposed to cold climate, especially those working in farming, forestry, industry, or construction (Makinen et al. 2006). The same pattern was seen in the present study. The anthropometric data, tobacco use, and disease spectrum in our cohort (Table 1) roughly corresponded with other recent Swedish investigations (Backman et al. 2014; Eriksson et al. 2010; Lindberg et al. 2006), which indicates that our study has included a representative sample of the population. Other authors have reported that frostbite is common in circumpolar areas (Ikaheimo and Hassi 2011), with a prevalence of serious frostbite of 1.5% for men and 0.5% for women in Finland (Raatikka et al. 2007), which is similar to our results. Regarding sensory hand symptoms, a recently published study on heavily cold-exposed Swedish military conscripts showed a significant decrease in sensory perception after winter training, which was evident even among those without any reported cold injury (Carlsson et al. 2016). The prevalence of Raynaud’s phenomenon in our study was high compared to previous studies on a general population, especially among men. In a recent review, the prevalence of Raynaud’s phenomenon was estimated to be 0.8–6.5% in men and 2.1–5.8% in women, with the exact figure depending on the region in which the study was conducted and how a case was defined (Garner et al. 2015). However, in the aforementioned Finnish study (Raatikka et al. 2007), the prevalence of Raynaud’s phenomenon was 11.9% for men and 12.4% for women, which compares well with our results showing a occurrence of 11.0% for men and 14.0% for women. Such a high occurrence might indicate secondary etiologies, such as hand-arm vibration injury or auto-immune diseases, but the cold climate in Sweden and Finland might also trigger Raynaud’s phenomenon and other hand symptoms more easily than in a warmer climate, a notion that is supported by epidemiological data (Block and Sequeira 2001).

There are several limitations to our study. The cross-sectional design does not allow causal relations to be established. The rather low response rate (35.9%) may limit the generalizability of the results and increase the uncertainty in prevalence estimates. There is also the possibility that symptomatic subjects might be more prone to respond to a questionnaire of this kind, and this might lead to an overestimation of both exposure and symptoms. The number of retired respondents was high, which might weaken any possible associations with occupational factors. The study region comprises a large area with a mean monthly temperature during the study period that spanned from about −9 to 5 °C, meaning that there is reason to suspect a variance in ambient cold exposure that has not been adjusted for in the analyses. Thus, the results in our study serve as an indication of health effects due to cold exposure in northern Sweden, but must be cautiously interpreted with regard to these limitations in study design and response rate.

Despite these limitations, this is the first population-based study on cold exposure and related health effects in Sweden, and it includes more than 12,000 participants. Despite the rather low response rate, the high occurrence of cold-related symptoms in the cohort still indicates that cold exposure poses a significant negative impact on health in northern Sweden. Our analysis revealed no major differences between responders and non-responders regarding geographical region, which was the expected main determinant of the cold exposure variables (Online Resource 1). Thus, we believe that the present study has included a representative sample of the population and that the possible bias produced by a low response rate has not affected the exposure data to any great extent. The cold exposure in our study was estimated from a subjective standpoint, but the close association between cumulative cold exposure and the occurrence of frostbite (which we consider as a definitive marker of cold exposure) supports that this approach to collecting exposure data was relevant (Table 3). This first study within CHINS will provide a baseline cohort for questionnaire-based case–control studies focusing on different cold-related health effects.

## Conclusion

The present study demonstrates that the cold environment in northern Sweden might be an underestimated health risk. Our hypothesis that cold exposure is positively related to reporting of neurovascular hand symptoms was supported by our findings. In addition, such symptoms were common not only in conjunction with an overt cold injury. Our results warrant further study on pathophysiological mechanisms and suggest the need for confirmatory prevalence studies to support national public health planning.

## Electronic supplementary material

Below is the link to the electronic supplementary material.

Supplementary material 1 (PDF 130 kb)

## References

[CR1] Backman H, Hedman L, Jansson SA, Lindberg A, Lundback B, Ronmark E (2014). Prevalence trends in respiratory symptoms and asthma in relation to smoking—two cross-sectional studies ten years apart among adults in northern Sweden. World Allergy Organ J.

[CR2] Baldus S, Kluth K, Strasser H (2012). Order-picking in deep cold—physiological responses of younger and older females. Part 2: body core temperature and skin surface temperature. Work.

[CR3] Block JA, Sequeira W (2001). Raynaud’s phenomenon. Lancet.

[CR4] Carlsson IK, Rosen B, Dahlin LB (2010). Self-reported cold sensitivity in normal subjects and in patients with traumatic hand injuries or hand-arm vibration syndrome. BMC Musculoskelet Disord.

[CR5] Carlsson D, Pettersson H, Burstrom L, Nilsson T, Wahlstrom J (2016). Neurosensory and vascular function after 14 months of military training comprising cold winter conditions. Scand J Work Environ Health.

[CR6] Eriksson M, Holmgren L, Janlert U, Jansson JH, Lundblad D, Stegmayr B (2010). Large improvements in major cardiovascular risk factors in the population of northern Sweden: the MONICA study 1986-2009. J Int Med.

[CR7] Garner R, Kumari R, Lanyon P, Doherty M, Zhang W (2015). Prevalence, risk factors and associations of primary Raynaud’s phenomenon: systematic review and meta-analysis of observational studies. BMJ Open.

[CR8] Gasparrini A, Guo Y, Hashizume M, Lavigne E, Zanobetti A, Schwartz J (2015). Mortality risk attributable to high and low ambient temperature: a multicountry observational study. Lancet.

[CR9] Hagberg M, Burstrom L, Lundstrom R, Nilsson T (2008). Incidence of Raynaud’s phenomenon in relation to hand-arm vibration exposure among male workers at an engineering plant a cohort study. J Occup Med Toxicol.

[CR10] Hassi J, Rytkonen M, Kotaniemi J, Rintamaki H (2005). Impacts of cold climate on human heat balance, performance and health in circumpolar areas. Int J Circumpolar Health.

[CR11] Hope K, Eglin C, Golden F, Tipton M (2014). Sublingual glyceryl trinitrate and the peripheral thermal responses in normal and cold-sensitive individuals. Microvasc Res.

[CR12] Ikaheimo TM, Hassi J (2011). Frostbites in circumpolar areas. Glob Health Action.

[CR13] Imray CH, Castellani JW, Auerbach PS (2012). Nonfreezing Cold-Induced Injuries. Wilderness Medicine.

[CR14] Imray CH, Grieve A, Dhillon S, The Caudwell Xtreme Everest Research Group (2009). Cold damage to the extremities: frostbite and non-freezing cold injuries. Postgrad Med J.

[CR15] International Labour Organization (2012) International Standard Classification of Occupations (ISCO-08), Geneva, Switzerland. ISBN 978-92-2-125953-4

[CR16] International Organization for Standardization (2008) ISO 15743:2008—Ergonomics of the thermal environment—Cold workplaces—Risk assessment and management, Brussels

[CR17] Kay S (1985). Venous occlusion plethysmography in patients with cold related symptoms after digital salvage procedures. J Hand Surg Br.

[CR18] Keim SM, Guisto JA, Sullivan JB (2002). Environmental thermal stress. Ann Agric Environ Med.

[CR19] Lindberg A, Bjerg A, Ronmark E, Larsson LG, Lundback B (2006). Prevalence and underdiagnosis of COPD by disease severity and the attributable fraction of smoking: report from the obstructive lung disease in Northern Sweden studies. Respir Med.

[CR20] Lithell M, Backman C, Nystrom A (1997). Pattern recognition in post-traumatic cold intolerance. J Hand Surg Br.

[CR21] Mackiewicz Z, Piskorz A (1977). Raynaud’s phenomenon following long-term repeated action of great differences of temperature. J Cardiovasc Surg (Torino).

[CR22] Makinen TM, Hassi J (2002). Usability of isothermal standards for cold risk assessment in the workplace. Int J Circumpolar Health.

[CR23] Makinen TM, Hassi J (2009). Health problems in cold work. Ind Health.

[CR24] Makinen TM, Raatikka VP, Rytkönen M, Jokelainen J, Rintamäki H, Ruuhela R (2006). Factors affecting outdoor exposure in winter: population-based study. Int J Biometeorol.

[CR25] Makinen TM, Jokelainen J, Näyhä S, Laatikainen T, Jousilahti P, Hassi J (2009). Occurrence of frostbite in the general population—work-related and individual factors. Scand J Work Environ Health.

[CR26] Nayha S, Hassi J, Jousilahti P, Laatikainen T, Ikaheimo TM (2011). Cold-related symptoms among the healthy and sick of the general population: national FINRISK Study data, 2002. Public Health.

[CR27] Negro C, Rui F, D’Agostin F, Bovenzi M (2008). Use of color charts for the diagnosis of finger whiteness in vibration-exposed workers. Int Arch Occup Environ Health.

[CR28] Raatikka VP, Rytkonen M, Nayha S, Hassi J (2007). Prevalence of cold-related complaints, symptoms and injuries in the general population: the FINRISK 2002 cold substudy. Int J Biometeorol.

[CR29] Swedish metrological and hydrological institute (2015) National Weather Data Report. www.smhi.se/klimatdata/meteorologi/temperatur. Accessed 20 Dec 2015

[CR30] Swedish Work Environment Authority (2014) The Work Environment 2013, Report no. 2014:3, Stockholm: Swedish Work Environment Authority

[CR31] The Eurowinter Group (1997). Cold exposure and winter mortality from ischaemic heart disease, cerebrovascular disease, respiratory disease, and all causes in warm and cold regions of Europe. Lancet.

[CR32] The Swedish horticultural society (2015) Climate zones for gardening. www.tradgard.org/svensk_tradgard/zonkartan.html. Accessed 11 Jan 2015

[CR33] Thomas JR, Oakley HN, Pandoff KB, Burr RE (2002). Nonfreezing cold injury. Medical aspects of harsh environments, textbooks of military medicine.

