# Supplementary material for: Neurovascular hand symptoms in relation to cold exposure in northern Sweden: a population-based study
Source: Int Arch Occup Environ Health. 2017 Apr 11;90(7):587–95. doi: 10.1007/s00420-017-1221-3 (PMC5583276; doi:10.1007/s00420-017-1221-3)
Supplement: Supplementary file 1 — Supplementary material 1 (PDF 130 kb) [file 420_2017_1221_MOESM1_ESM.pdf]

Article title:
Neurovascular hand symptoms in relation to cold exposure in northern Sweden – a population-based study

Journal name:
International Archives of Occupational and Environmental Health

Author names:
A Stjernbrandt[1], B Björ[1], M Andersson[1], L Burström[1], I Liljelind[1], T Nilsson[1], R Lundström[1,2], J Wahlström[1]

Affiliations:
[1]Department of Public Health and Clinical Medicine, Umeå University, 901 87 Umeå, Sweden
[2]Department of Radiation Sciences, Umeå University, 901 87 Umeå, Sweden

Corresponding author:
Dr. Albin Stjernbrandt
Occupational and Environmental Medicine
University Hospital of Umeå
901 85, Umeå
Tel +46 90 785 99 52
Fax +46 90 785 24 56
albin.stjernbrandt@umu.se

Online Resource 1. Non-responder analysis

Non-responders and responders have been stratified according to sex, county, and age.

		Non-responders		Responders	
		N	%	N	%
**County**					
Norrbotten	♂	3190	69.0	1436	31.0
	♀	2718	61.8	1679	38.2
Västerbotten	♂	3733	67.2	1824	32.8
	♀	2934	58.1	2120	41.9
Västernorrland	♂	3080	68.4	1425	31.6
	♀	2606	59.4	1778	40.6
Jämtland	♂	2356	69.1	1056	30.9
	♀	1847	58.5	1309	41.5
**Age group [years]**					
18–30	♂	3908	31.6	717	12.5
	♀	3130	31.0	965	14.0
31–45	♂	2943	23.8	857	14.9
	♀	2437	24.1	1230	17.9
46–60	♂	3027	24.5	1490	26.0
	♀	2406	23.8	1893	27.5
61–70	♂	2481	20.1	2677	46.6
	♀	2132	21.1	2798	40.6
		N	SD	N	SD
**Mean age**					
	♂	41.3	(15.0)	51.6	(14.6)
	♀	41.6	(15.2)	49.8	(14.6)
